# Supplementary material for: Indoxyl Sulfate Contributes to mTORC1-Induced Renal Fibrosis via The OAT/NADPH Oxidase/ROS Pathway
Source: Toxins (Basel). 2021 Dec 18;13(12):909. doi: 10.3390/toxins13120909 (PMC8706756; doi:10.3390/toxins13120909)
Supplement: Supplementary file 1 [file toxins-13-00909-s001.zip › toxins-1488738.pdf]

Supplementary Materials: Indoxyl sulfate contributes to mTORC1-induced renal fibrosis *via* the OAT/NADPH/oxidase/ROS pathway

Takehiro Nakano, Hiroshi Watanabe, Tadashi Imafuku, Kai Tokumaru, Issei Fujita, Nanaka Arimura, Hitoshi Maeda, Motoko Tanaka, Kazutaka Matsushita, Masafumi Fukagawa, and Toru Maruyama

SUPPLEMENTARY METHODS

Biochemical evaluation of blood samples

The plasma levels for BUN and creatinine were measured by a FUJI DRI-CHEM 7000 and DRI-CHEM slides system (FUJIFILM, Tokyo, Japan) following the manufacturer's protocol. Red blood cell count and hemoglobin levels were measured using an automatic hematology analyzer (KX-21NV; Sysmex, Kobe, Japan). The values for the biochemical evaluation in plasma from CKD mice are listed in Supplementary Table 1.

ROS measurements

HK-2 cells were seeded on 96-well plates at 1.0×10^4 cell per well. After removing the culture solution, CM-H_2DCFDA was added and the resulting prapartion was allowed to become incorporated into cells by incubating at 37°C for 30 min. After removing the supernatant and adding D-PBS or uremic toxins (1 mM) the fluorescence intensity was measured with a fluorescence plate reader (excitation/emission = 485 nm/535 nm, Spectra Fluor, TECAN).

SUPPLEMENTARY RESULTS

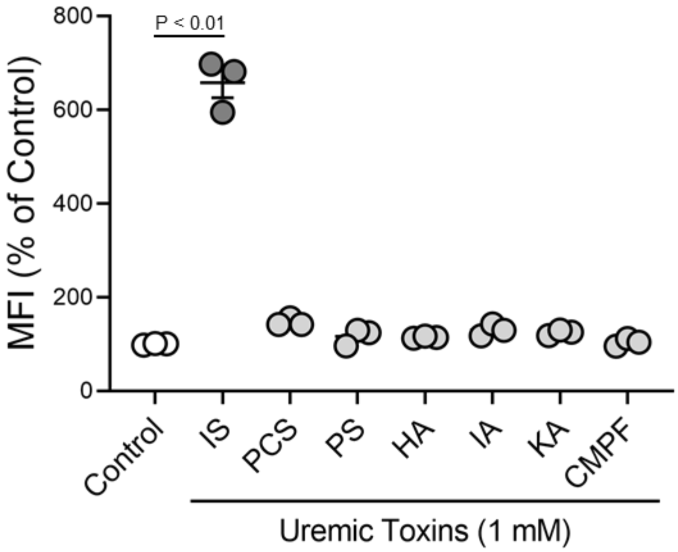

Figure S1. Effect of seven uremic toxins on the production of ROS in HK-2 cells. HK-2 cells were starved by incubation in serum-free medium for 2 hours and then treated with CM-H2DCFDA in PBS for 30 min. After the removal of the D-PBS, the cells were treated with each uremic toxin (1 mM) and incubated for 90 min.

Fluorescence intensity was measured at an excitation wavelength of 485 nm and at an emission wavelength of 535 nm. Data are expressed as the mean ± SEM (n = 3).

SUPPLEMENTARY TABLES

Table S1. The list of antibodies for Western blotting (upper table) and primers for quantitative RT-PCR (lower table).

Primary Antibody	Company	Catalog #	Species	Dilution ratio	Working Solution
p-S6	Cell Signaling Technologies	4858	Rabbit	1:2000	1% skim milk in TBS-T
S6	Cell Signaling Technologies	2217	Rabbit	1:1000	1% skim milk in TBS-T
E-cadherin	R&D Systems	AF648	Goat	1:2000	1% skim milk in TBS-T
α-SMA	Abcam	Ab5694	Rabbit	1:2000	1% skim milk in TBS-T
COL1A1	Cell Signaling Technologies	84336s	Rabbit	1:1000	1% skim milk in TBS-T
β-actin	Sigma	A5316	Mouse	1:2000	1% skim milk in TBS-T
**Secondary Antibody**	**Company**	**Catalog #**	**Species**	**Dilution ratio**	**Working Solution**
anti-rabbit IgG-HRP	Santa Cruz Biotechnology	sc-2357	Rabbit	1:5000	1% skim milk in TBS-T
anti-mouse IgGκ BP-HRP	Santa Cruz Biotechnology	sc-516102	Mouse	1:5000	1% skim milk in TBS-T
anti-goat IgG-HRP	Santa Cruz Biotechnology	sc-2768	Rabbit	1:5000	1% skim milk in TBS-T

Primer Name	Forward Primer Sequence (5′-3′)	Reverse Primer Sequence (5′-3′)
Human IL-6	CAGTTCCTGCAGAAAAAGGC	AACAACAATCTGAGGTGCCC
Human TNF-α	TGAAAGCATGATCCGGGACG	CAGCTTGAGGGTTTGCTACAAC
Human GAPDH	GGTGAAGGTCGGAGTCAACG	ACCATGTAGTTGAGGTCAATGAAGG
Mouse IL-6	TCTCTGCAAGAGACTTCCATCC	AGACAGGTCTGTTGGGAGTG
Mouse TNF-α	CATGAGCACAGAAAGCATGATCCG	AAGCAGGAATGAGAAGAGGCTGAG
Mouse GAPDH	AACTTTGGCATTGTGGAAGG	ACACATTGGGGGTAGGAACA

Table S2. Plasma biochemical parameters in CKD mice. BUN, SCr, RBC, and Hb for control mice and 0.2% adenine-containing diet (CKD) feeding mice without/with AST-120 or rapamycin treatments.

	**Control**	**CKD**	**CKD + AST-120**	**CKD + Rapamycin**
BUN (mg/dL)	27.8 ± 2.8	40.6 ± 4.3 [a]	36.9 ± 1.9 [a]	36.9 ± 2.1 [a]
SCr (mg/dL)	0.18 ± 0.04	0.29 ± 0.04 [a]	0.26 ± 0.05 [a]	0.26 ± 0.06 [a]
RBC (×10^4/μL)	746 ± 16	613 ± 8 [a]	700 ± 9 [b]	695 ± 21 [b]
Hb (g/dL)	12.0 ± 0.1	9.7 ± 0.2 [a]	11.1 ± 0.4 [b]	11.1 ± 0.5 [b]

Data are expressed as the mean ± SE (n = 5).

[a] $p < 0.05$ compared with Control.

[b] $p < 0.05$ compared with CKD.

BUN, blood urea nitrogen; SCr, serum creatinine; RBC, red blood cell; Hb, hemoglobin
